# Conceptual Framework of Health-Literate Nursing System: A Proceduralized Grounded Theory Approach

**DOI:** 10.1155/jonm/1496009

**Published:** 2025-07-18

**Authors:** Yixue Wu, Yunxia Li, Lu Liu, Qiuhuan Huang, Yaping Feng, Jianyi Wang, Yanghuan Zhou, Lan Yao, Jianmei Xia, Huina Xu, Meng Yang, Lihui Gu, Wenbo Qiu, Yingge Tong

**Affiliations:** ^1^School of Public Health and Nursing, Hangzhou Normal University, Hangzhou, China; ^2^Nursing Department, The Affiliated Lihuili Hospital of Ningbo University, Ningbo, China; ^3^Nursing Department, Sir Run Run Shaw Hospital, Zhejiang University School of Medicine, Hangzhou, China; ^4^Affiliated Hospital of Youjiang Medical University for Nationalities, Guangxi Zhuang Autonomous Region, Baise, China; ^5^Guangxi Clinical Medical Research Center for Hepatobiliary Diseases, Guangxi Zhuang Autonomous Region, Baise, China; ^6^Nursing Department, The Affiliated Hospital of Hangzhou Normal University, Hangzhou, China; ^7^Department of Nursing, Hubei Cancer Hospital, Wuhan, China; ^8^Department of Cardiothoracic Surgery, Taizhou Hospital of Zhejiang Province, Taizhou, China; ^9^Ward 3, Department of Oncology, Hangzhou Cancer Hospital, Hangzhou, China; ^10^Department of Nursing, The First Hospital of Jiaxing, Jiaxing, China; ^11^Administration Office, Hangzhou Xixi Hospital, Hangzhou, China; ^12^Department of Neonatology, Taizhou Municipal Hospital, Taizhou, China

## Abstract

**Aim:** To develop and define a conceptual framework for a system-level nursing approach that addresses patients' health literacy needs.

**Background:** Health literacy has evolved beyond individual-level concerns to system-level responsibilities. While several frameworks, such as the Health Literate Healthcare Organization and Organizational Health Literacy, have guided institutional reforms, they often neglect the unique functions and contributions of the nursing system. Hence, a need exists for clear, structured guidance to integrate health literacy into nursing practice and management.

**Methods:** A proceduralized grounded theory approach was employed. In-depth interviews were conducted with 23 healthcare professionals from diverse institutions between August 2021 and May 2022. Field observations were performed in one tertiary and one secondary hospital from December 2021 to March 2022. Data were analyzed through open, axial, and selective coding. Theoretical saturation was confirmed with additional interviews conducted between July and September 2024.

**Results:** A conceptual framework, designated the Health-Literate Nursing System (HLNS), was developed. HLNS centers on health literacy–oriented nursing support, comprising five interrelated elements: (1) nurses who lead health literacy promotion, (2) patients who participate in health literacy initiatives with nursing support, (3) a health-literate nursing material environment, (4) a health-literate nursing management system, and (5) a health-literate external system. These components synergistically facilitate clear communication, equitable access to nursing services, and health information dissemination.

**Conclusions:** This study proposes a conceptual framework for the HLNS, offering a system-level approach to enhancing healthy literacy in patients and nursing systems. It emphasizes nurse leadership, patient engagement, supportive material environments, effective management structures, and cross-sector collaboration.

**Implications for Nursing Management:** The HLNS framework offers practical guidance for nursing leaders to advance system-level health literacy. It underscores the importance of resource allocation, workforce development, interdepartmental collaboration, and standardized tools to assess and improve nursing system performance.

## 1. Introduction

Health literacy has emerged as a pressing global concern [1]. Individuals with low health literacy often struggle to access, evaluate, and apply health information and services to maintain or improve their quality of life [2], potentially experiencing adverse health-related outcomes [2, 3]. They are less capable of managing chronic diseases and face greater difficulty communicating with medical staff and engaging in shared decision-making compared to individuals with adequate health literacy [4, 5].

Health literacy is not solely a personal attribute but also a product of healthcare system complexity [6–8]. Even individuals with high functional literacy may struggle to access and navigate care when systems are fragmented or inaccessible [9]. Accordingly, health literacy is recognized as an organizational attribute, requiring institutions to minimize literacy-related barriers and create accessible environments that help individuals access and apply health information and services [10, 11]. The governments of the United States [1] and Australia [12], along with the World Health Organization (WHO) [13], have shifted their focus toward system-level strategies to simplify healthcare and address individual health literacy needs.

Several concepts have been proposed to address health literacy challenges. The Institute of Medicine (IOM) [14] introduced Health Literate Healthcare Organization (HLHO) as “health care organizations that make it easier for people to navigate, understand, and use information and services to take care of their health.” Similarly, the U.S. Department of Health and Human Services [1] defined Organizational Health Literacy (OHL) in Healthy People 2030 as “the degree to which organizations equitably enable individuals to find, understand, and use information and services to inform health-related decisions and actions.” Additionally, the concept of Organizational Health Literacy Responsiveness (Org-HLR) [15] referred to “the ability of health organizations and systems to recognize and respond to health literacy needs and improve health environments to ensure equitable access to and use of information and services”.

These conceptual frameworks have driven institutional transformation by prompting healthcare organizations to prioritize accessibility, clarity, and responsiveness in health information and service delivery. Vellar et al. [16] applied the HLHO model to integrate health literacy principles into healthcare systems, improving patient engagement. Similarly, interventions have promoted the use of plain language among staff and enhanced the clarity of health information [12, 17]. These initiatives have reinforced organizational commitment and integrated health literacy principles into institution-wide policies and practices [18].

Despite these advances, current frameworks predominantly address system-level change from a general institutional perspective, neglecting the pivotal role of nurses. Nurses comprise nearly half the global health workforce. They serve as frontline communicators and educators in patient care [19], uniquely equipped to deliver personalized education, encourage health-promoting behaviors, and advocate for patients' needs [20]. To support these functions, nursing systems must incorporate health literacy principles. However, existing models fail to clearly define nurses' responsibilities in promoting health literacy or provide a framework tailored to the operational realities of nursing systems.

From a nursing management perspective, many system-level health literacy reforms, such as those targeting hospital accessibility or health insurance policy, extend beyond the nursing domain. Thus, nursing management should prioritize meso-level reforms that align with nursing operations. This includes enhancing the quality of patient education, streamlining nursing workflows, and optimizing the dissemination of information [21, 22]. Nurses are also vital in simplifying care by helping patients navigate services, interpret health information, and coordinate with other providers. These roles are consistent with the core principles of OHL, which aim to make healthcare more accessible and understandable. However, current system-level health literacy frameworks rarely specify nurses' contributions or offer guidance for developing health-literate nursing systems (HLNSs).

Research on OHL in China remains limited [23]. To date, only Tong and colleagues [24] have adapted the OHL framework to the Chinese context, while its relevance to nursing practice has not been thoroughly explored. Considering the pivotal role of nurses in promoting health literacy, there is a pressing need to conceptualize a system-level nursing approach that establishes a supportive environment, enabling nurses to lead health literacy efforts and empowering patients to access and utilize health-related information and nursing services effectively.

This study aimed to conceptualize such an approach by developing a set of criteria for system-level nursing strategies to address health literacy in China. The primary research questions were: (1) How should system-level nursing approaches for addressing health literacy needs be defined? and (2) What constitutes a supportive environment facilitating health literacy in nursing practice?

## 2. Materials and Methods

### 2.1. Design

A proceduralized grounded theory approach—emphasizing the immersion of researchers into the study context and collecting data from those deeply involved [25, 26]—was adopted to construct an explanatory model. This approach supports the generation of context-sensitive concepts that can explain and predict the phenomenon under investigation [27]. Unlike traditional grounded theory, this method encourages using existing literature and theories, aiding in logical thinking and facilitating the discovery of causal relationships [28]. Its structured steps ensure analytical rigor, reliability, and iterative verification, fostering a robust theoretical framework. Proceduralized grounded theory is particularly well suited for conceptual development in fields with emerging theoretical understanding [27]. Given that system-level health literacy remains undertheorized in nursing, this study applied the health literacy tapestry model (HLTM) and system management theory as guiding frameworks.

#### 2.1.1. HLTM

HLTM, proposed by Parnell [29], employs a holistic nursing approach whereby nurses not only enhance individual health literacy but also consider environmental factors such as physical conditions and social support that impact patients' abilities to acquire, understand, and utilize health information and services. HLTM asserts that an individual's level of health literacy is influenced by the complexity of the specific environment. The model identifies six antecedents affecting individual and system/provider factors: “demographics,” “overall health status,” “health knowledge and experience,” “community support,” “cultural, spiritual and social influences,” and “media and marketplace.” The first three represent individual factors, while the latter three capture external environmental factors. Additionally, Parnell emphasized that promoting health literacy fundamentally involves addressing misconceptions and unconscious biases between healthcare providers and the population they serve.

#### 2.1.2. Systems Management Theory

Systems management theory, developed by Kast and Rosenzweig [30] based on the general systems theory, has been widely applied in healthcare. It views a system as an organized whole of interacting components within an environment. Key characteristics of the system include: openness to surrounding environmental influences (e.g., media and government); hierarchical structure with subsystems that function efficiently to achieve the requirements of the higher-level systems; integrity and intercoordination of subsystems to improve the overall performance, creating a synergistic effect where “1 + 1 > 2”; and purposeful activities aimed at specific functions.

Based on the systems management theory, Jiang [31] proposed that the nursing system, characterized by its complex structure, comprises subsystems such as clinical nursing, nursing education, and nursing management. This system is a crucial element of healthcare and integrates new information, technologies, and equipment from external sources while interacting with and being constrained by healthcare and social systems. Nurses and patients serve as central components within this system, forming the basis for internal processes and external interactions.

By integrating HLTM with systems management theory, this study conceptualizes health literacy as the product of the dynamic interplay between individual skills and environmental factors. The nursing system is perceived as an open system that positively interacts with external systems while ensuring the efficient operation of internal subsystems. Furthermore, the system facilitates the provision of clear health information and accessible nursing services to patients. Nurses, those leading and participating in health literacy initiatives, are considered pivotal to this system-level interaction.

**Figure 1:**
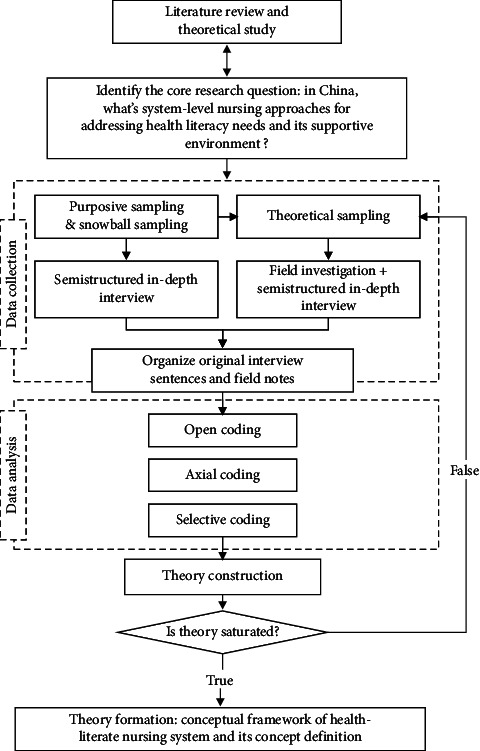
Framework of study design.

The study was conducted in three stages (Figure 1). First, semistructured in-depth interviews explored how the nursing system within the healthcare organization facilitated patients' ability to access, understand, and use health information and nursing services. Second, field investigations supplemented the data that could not be obtained from the interviews. Finally, open, axial, and selective coding was applied to determine the core elements of nursing-based, system-level approaches to addressing health literacy. A conceptual framework and new concept were defined from these findings.

### 2.2. Sample

The study's sampling involved in-depth interviews and field investigations. The interview sample was selected by combining purposive, snowball, and theoretical sampling. To ensure representation, participants were required to have at least 5 years of experience in healthcare organization management, clinical practice, or health management. Through purposive sampling, nursing department directors, head nurses, and bedside nurses with substantial clinical experience were recruited from various healthcare institutions. Initial participants then recommended others via snowball sampling.

Early data collection and coding revealed that system-level nursing approaches for addressing health literacy needs was influenced by hospital-level strategic planning, national health policy, interprofessional collaboration, and digital infrastructure. As categories, such as “Health-Literate External Systems” and “Construction of Network Information Systems,” emerged, theoretical sampling recruited additional participants, including hospital managers, clinical physicians, and health administration officials, to saturate these categories and refine the conceptual framework.

Field investigations supplemented interview data. One tertiary and one secondary hospital were selected as observation sites. In China, healthcare organizations are classified into tertiary, secondary, or primary levels based on their capacity to provide medical services, education, and research [32, 33]. Tertiary and secondary hospitals offer comprehensive medical services, while primary hospitals focus on preventive care. Given the limited scope of clinical services in primary hospitals, they were excluded from this study. Descriptive and focused observational methods were used to examine interactions among outpatients and inpatients and nursing practices across different departments.

### 2.3. Data Collection

An interview guide containing key open-ended questions was developed based on the methodological principles of the Problem-centered Interview approach. The guide was reviewed by a health literacy expert and pilot-tested with two bedside nurses and one head nurse before finalization. The main questions included: (1) What nursing measures, equipment, or facilities should be employed to help patients more easily and efficiently find, understand, and use health information and access nursing services? (2) What internal and external factors within healthcare organizations influence patients' ability to access, comprehend, and use health information and nursing services? (3) What changes occur in patients and nurses when nursing system for health literacy promotion are fully implemented?

The first author conducted all interviews between August 2021 and May 2022. Participants were scheduled in advance and interviewed one to three times in a quiet, uninterrupted setting. Each interview lasted approximately 40 to 50 min. All sessions were audio recorded and transcribed verbatim. Transcripts from the initial interviews were returned to participants via WeChat, or email for member verification to ensure the accuracy and authenticity of the content. Some participants offered additional insights or raised new ideas during this process, prompting follow-up interviews. Additional interviews were conducted to clarify ambiguous responses or further explore emerging themes.

The first author conducted field investigations between December 2021 and March 2022. The researcher, a registered female nurse with a master's degree in nursing, has thorough knowledge of the study topic and expertise in interview and observation techniques. Field investigations were carried out to gather supporting information not captured during interviews. (1) Four patients (two with low and two with adequate health literacy) were observed throughout their hospitalization, focusing on nurse–patient communication in high-risk situations (e.g., medication administration, preoperative education). Observations targeted communication strategies and barriers patients encountered when accessing information or services. Informed consent was obtained from all observed patients. (2) The format and content of health information materials provided by the nursing departments (or nursing units) and the availability of related resources were reviewed. Institutional documents and policies issued by nursing departments or hospitals that guide nurses in addressing patients' health literacy, promoting clear nurse–patient communication, and improving the accessibility of nursing services were collected. Data comprised field notes, memos, and photographs organized on the same day to form a complete observational record. To enhance reliability, data were triangulated through informal interviews with clinical nurses, patients, and caregivers. The researcher verified the content with the observed nurses and patients to ensure authenticity.

Data collection was anonymous, with participants designated as P1, P2, etc., and field observations as F1 and F2.

### 2.4. Ethical Considerations

Ethical approval was granted by the Institutional Review Board of the School of Public Health and Nursing, Hangzhou Normal University, China (Grant ID: 2022031). The participants were informed of the study's purpose, objectives, and methods and provided written informed consent.

### 2.5. Data Analysis

Data analysis was conducted concurrently with data collection, following the proceduralized grounded theory approach. Three sequential coding stages were employed: open, axial, and selective [34, 35].

During open coding, data were examined line by line to extract meaningful segments. These segments were labeled and analyzed using the constant comparative method to conceptualize initial codes, which were compared, clustered, and refined into categories.

In axial coding, the relationships among categories were examined to identify main and subcategories. In selective coding, a core category was identified from the major categories, forming the basis for a theoretical understanding of system-level health literacy promotion in nursing. Subsequently, theoretical sampling refined unsaturated categories and revised emerging concepts. Finally, the intrinsic relationships and causal connections of the core and other categories were analyzed, constructing the concept and clarifying the logical framework.

A coding team comprising three researchers was formed to minimize subjective bias and enhance credibility [36]. Led by Researcher T, an associate professor with expertise in qualitative research, the team included Researchers W and X, trained in proceduralized grounded theory and familiar with the study domain. They independently coded interview transcripts and field notes to create the initial coding framework. The coding team met to discuss discrepancies and reach consensus. During the axial and selective coding, additional data were collected if certain categories lacked sufficient supporting data or required clarification, and previous coding steps were refined.

### 2.6. Rigor

Several strategies were employed to ensure the study's rigor. First, interview participants were purposefully selected to represent diverse characteristics, including age, educational background, and professional position. Second, interview recordings were transcribed within 24 h to enhance data credibility and returned to participants for member verification (Supporting 1). Additionally, informal interviews with nurses and patients were conducted to verify field note accuracy.

All members of the coding team were familiar with the research context and received formal training in proceduralized grounded theory. Associate Professor T., an expert in qualitative research, oversaw the coding process and was responsible for resolving coding discrepancies and finalizing coding results.

To assess theoretical saturation, ten healthcare professionals not involved in the initial phase of the study were interviewed between July and September 2024. This follow-up assessment was designed to test the robustness and temporal consistency of the theoretical categories derived from the 2021 and 2022 data. No new categories or relationships emerged, indicating theoretical saturation was achieved (Supporting 2).

## 3. Results

Interviews were conducted between August 2021 and May 2022 with 23 participants recruited from secondary and tertiary hospitals (*n* = 16, 69.6%), primary hospitals (*n* = 5, 21.7%), and health administrative departments (*n* = 2, 8.7%; Table 1). Among them were bedside nurses (*n* = 9, 39.1%), head nurses (*n* = 3, 13.0%), directors of nursing departments (*n* = 4, 17.4%), clinical doctors (*n* = 2, 8.7%), administrative staff from healthcare organizations (*n* = 3, 13.0%), and officers from health-administrative government departments (*n* = 2, 8.7%). Participants from hospitals (*n* = 21) held junior (*n* = 3; 14.3%), intermediate (*n* = 7; 33.3%), or senior professional titles (*n* = 11; 52.4%). The two officers from health administrative government departments were a Level IV Division Rank official and Level II Principal Staff Member. The average years of work experience among the participants was 13.26 ± 6.04 years.

Field investigation was conducted in two hospitals in Zhejiang Province: one secondary hospital (ID: F1) and one tertiary hospital (ID: F2).  Table 2 presents the demographic information of the field sites.

A total of 254 codes were identified and divided into 28 categories (Supporting 3), which were consolidated into 14 subcategories and subsequently into five main categories. Among them, health literacy–oriented nursing support was selected as the core category (Table 3). The five main categories were (1) nurses who lead health literacy promotion, (2) patients who participate in health literacy initiatives with nursing support, (3) a health-literate nursing material environment, (4) health-literate nursing management system, and (5) health-literate external system. Based on these results, a conceptual framework of the HLNS was developed, and the concept was formally defined.

### 3.1. Nurses Who Lead Health Literacy Promotion

Nurses were identified as the primary drivers of health literacy promotion within healthcare organizations. This main category highlights the leading role nurses play in designing and implementing communication strategies that address patients' varying health literacy levels. These efforts aim to enhance the accessibility of health information and support patients in effectively utilizing nursing services. Three subcategories emerged under this theme: clear nurse–patient communication, meeting the needs of patients with low health literacy, and inter-nurse collaboration.

#### 3.1.1. Clear Nurse–Patient Communication

The first subcategory focuses on communication practices adopted by nurses to ensure patient understanding. Interviewees reported that many patients were hesitant to ask questions during interactions, posing a significant challenge to effective communication. To address this, nurses assumed that all patients may have difficulty understanding health information and proactively adopted strategies such as simplified verbal explanations, written materials, visual aids, and videos to enhance comprehension.

**F1** (field investigation, observation nurse–patient communication): “*At the end of preoperative communication, nurses will ask patients to repeat the instructions, such as when they start fasting.*”

**P10** (interview): “*The guide sheet lists the scheduled time, precautions, and examination location. Nurses mark key points and orally emphasize them*.”

Another key component of clear nurse–patient communication is prioritizing of high-risk scenarios where misunderstanding may lead to adverse outcomes—such as preoperative consultations, medication instructions, and discharge planning. In these contexts, nurses adopted additional strategies to reinforce patient comprehension.

**P23** (interview): “*For breast cancer patients discharged from the hospital, we provide a personal treatment planner. It included a calendar with the next check-up or treatment date highlighted and lists the required tests*.”

#### 3.1.2. Meeting the Needs of Patients With Low Health Literacy

The second subcategory relates to identifying and supporting patients with limited health literacy. More than half of the participants agreed that nurses should utilize specific assessment tools to screen for low health literacy. However, many noted that such tools are not yet routinely implemented in clinical settings. Consequently, experienced nurses often relied on their clinical judgment and observation. Common indicators used to identify patients with low health literacy include older age (typically ≥ 60 years), low educational level (elementary school or below), and requiring repeated explanations or extended communication to ensure comprehension.

**P4** (interview): “*Based on our personal experience, we assess the patient's learning ability and health literacy. According to these assessments, we choose appropriate communication methods during discussions about their condition or health education, ensuring that even those with low health literacy can understand.*”

Nurses delivered personalized assistance to patients with limited health literacy, ensuring respectful and supportive interactions without criticism or condescension.

**P3** (interview): “*When dealing with patients with low education levels, we typically use anatomical models or teaching aids to provide further demonstrations*.”

**P19** (interview): “*Even if older patients struggle to grasp the education fully, we do not neglect them. Instead, they repeatedly emphasize the key points to the patients and their families*.”

#### 3.1.3. Inter-Nurse Collaboration

The third subcategory highlights how nurses share health literacy–related information within their teams to ensure continuity of care. Participants described incorporating patients' health literacy levels into nursing handover reports, enabling colleagues to tailor communication accordingly. A shared commitment to “clear communication” was fostered across nursing teams, reinforcing a collaborative culture that supports patient-centered health literacy practices.

**F2** (field investigation, observation inter-nurse communication): “*After multiple education sessions, the patient still did not fully understand. During shift handovers or when transferring to another department, the nurse informed the incoming nurse about the patient's situation*.”

**P9** (interview): “*The teach-back method is part of skill assessments. Before assessments, I often ask my senior colleagues to point out any issues in my communication with patients because they have more experience*.”

### 3.2. Patients Who Participate in Health Literacy Initiatives With Nursing Support

When supported by nurses, patients played a crucial role in health literacy. With professional guidance, they engaged in medical decision-making, effectively accessed health information and services, and contributed to peer learning by sharing self-management experiences. Many participants also expressed appreciation for nurses' expertise in health education and coaching. This main category comprised three subcategories: promoting patient participation, earning recognition and trust, and guiding patients in accessing information and services.

#### 3.2.1. Nurses Promoting Patient Participation

Findings from interviews and field investigations indicated that while patients had intrinsic motivation for self-health management, their participation depended heavily on the facilitation of healthcare professionals. Nurses connected patients and medical teams, ensuring information was shared and facilitating patient involvement in medical decision-making. Additionally, they encouraged patients to participate in developing and evaluating health information. They also promoted patient involvement in peer education by sharing their self-management experiences.

**P23** (interview): “*There was a patient with early-stage breast cancer who struggled to decide whether to preserve her breast. After extended discussions, I learned that she leaned towards preservation but felt she lacked enough information. I helped her list her questions on paper and invited the doctor to assist those I could not answer. Only then did she make the best decision for herself*.”

**P1** (interview): “*After we create educational videos, we show them to patients for feedback. They are always willing to evaluate, often pointing out that the content is not simple enough or that the video is too long*.”

**F2** (field investigation, informal interview with a patient [male, 52 years]): “*I am a diabetic. The nurses often praise me for my good blood glucose management. I would love to accept the invitation to attend the presentation and share my diet control experience!*”

#### 3.2.2. Nurses Earning Recognition and Trust

Some bedside nurses reported challenges in health education stemming from patients' misconceptions about the nursing role—many perceived nurses as primarily responsible for technical tasks such as injections. In contrast, head nurses described smoother educational interactions, attributing their success to demonstrated expertise in health coaching. When nurses gained recognition as knowledgeable professionals, patients were more inclined to consult them, accept their advice, and adhere to their recommendations.

**P23** (interview): *“Some patients think nurses are just there to administer injections and distribute medications. They prefer to talk with doctors and follow doctors' advice, believing nurses' health guidance is not professional.”*

**P11** (interview)*: “Before starting health education, I clearly explain its purpose and provide some professional knowledge about the disease. When patients recognize your expertise, they naturally become more willing to listen.”*

#### 3.2.3. Nurses Guiding Patients in Accessing Information and Services

Field observations revealed patients often relied on unverified or misleading online videos and articles for health-related information. As a countermeasure, the participants emphasized the role of nurses in guiding patients to seek credible sources of healthcare information and services.

**F1** (field investigation, observation)*: “Patients often watch short videos of health knowledge, such as ‘Do this often, and you will never need a doctor' or ‘Five foods you should never eat together.' The video uploaders are typically nonmedical individuals. They dressed like a doctor with white coats, presenting themselves as medical authorities. Patients perceive these videos as authoritative medical advice.”*

**P23** (interview)*: “Patients discharged with new Peripherally Inserted Central Catheter (PICC) need to return for maintenance. I teach them how to use the hospital's WeChat official account to schedule PICC appointments or access online nursing consultations.”*

### 3.3. A Health-Literate Nursing Material Environment

The nursing material environment provides critical infrastructure that supports nurses in delivering health literacy–oriented care. Nursing administrative departments (i.e., hospital-level nursing offices) optimize health information resources and digital systems to improve communication and service delivery. Health materials must be accessible and understandable, supported by user-friendly platforms and technology. This main category comprises three subcategories: construction of nursing health information resources, development of network information systems, and material and financial support.

#### 3.3.1. Construction of Nursing Health Information Resources

Compared with materials developed for physicians, nursing health information resources were more practical, actionable, and tailored to diverse patient needs. To accommodate varying health literacy levels, content provided in multiple formats, such as videos, illustrated booklets, and simplified text, and placed in prominent, easily accessible locations within care environments. The goal is to empower patients to obtain and understand relevant information without professional assistance.

**P10** (interview): “*Given that everyone learns differently, we aim to design materials that combine text and visuals, making them easier for patients of various educational levels to review.”*

**F2** (field investigation, observation)*: “Printed materials are placed on prominent racks for patients to take for free. Digital materials are made accessible by posting QR codes on the walls of hospital wards, allowing patients to scan and access the information.”*

#### 3.3.2. Development of Network Information System

Nursing administrative departments have worked to optimize the digital information environment and expand access to online nursing services. Various “Internet + Nursing Services” initiatives have been launched, including online consultations for chronic disease management, maternal and infant nutrition guidance, and in-home nursing appointment scheduling. To support these services, multiple digital platforms, such as chat groups, WeChat mini-programs, and mobile applications, have been established to disseminate nursing-related health information and facilitate patient access.

**P1** (interview): *“Some of my patients can directly consult me about their PICC care through the hospital's official WeChat account, which is very convenient for them.”*

**P4** (interview)*: “Our department and hospital's WeChat official account or mobile app regularly push out nursing-related health education articles. We also create WeChat groups for inpatients or discharged patients to share rehabilitation exercise videos.”*

#### 3.3.3. Material and Financial Support

Nursing administrative departments have allocated material and financial resources to support health literacy initiatives. These include the provision of standardized, high-quality educational booklets, access to free video editing tools, and dedicated funding, allocated according to institutional budgets, to develop and implement health literacy promotion practices.

**P5** (interview)*: “For printed health booklets, our nursing administrative department provides a repository of materials for reference. For video production, free video editing toolkits are available for download.”*

**P11** (interview)*: “The funding for printing comes from the nursing administrative department's allocation.”*

**P9** (interview)*: “Winning competitions for designing nursing materials or awards for mentors in health literacy promotion could get a bonus.”*

### 3.4. A Health-Literate Nursing Management System

A health-literate nursing management system reflects a strategic priority within nursing leadership. It promotes health literacy by cultivating a supportive organizational culture, establishing relevant policies, and ensuring the allocation of essential human resources. This main category comprises three subcategories: strategic planning in nursing management, human resource allocation, and nursing quality improvement.

#### 3.4.1. Strategic Planning in Nursing Management

Strategic planning for health literacy within nursing management includes formal policy development and cultural integration. Nursing leaders are tasked with integrating the assessment and promotion of patient health literacy into institutional mandates. In practice, this includes the formation of dedicated committees to manage health literacy initiatives within the nursing system. Additionally, nursing managers advocate for a culture that prioritizes clear communication and patient-centered education.

**P8** (interview): “*The nursing administrative department director is a member of the health education committee. They are responsible for formulating health education and health literacy promotion institutions and organizing various health education activities*.”

**P19** (interview): “*We promote mentor nurses with exceptional health literacy promotion skills and publicize their achievements.*”

**P1** (interview): “*We required that nurses use strategies such as chunk & check and teach-back during nurse–patient communication to help patients fully and correctly understand health information*.”

#### 3.4.2. Human Resource Allocation

The nursing administrative department is critical in providing human resource support for health literacy promotion. This includes delivering targeted training and competency assessments while allocating nursing staff to clinical departments based on the complexity of patients' health education needs. By aligning workforce distribution with educational demands, the department ensures that health literacy initiatives are effectively implemented at the point of care.

**P8** (interview)*: “For newly hired nurses and interns, we focus on instilling the hospital culture of improving health literacy and raising their awareness of it. For senior nurses, the emphasis is on teaching them clear communication techniques, such as the teach-back method.”*

**P23** (interview): “*In our oncology department, we assign a nurse with extensive experience in health education as a health education specialist. She is specifically responsible for handling more complex or time-consuming health education tasks related to cancer, radiation, and chemotherapy*.”

### 3.5. A Health-Literate External System

The nursing administrative department has fostered collaboration across internal healthcare departments to create accessible patient environments and supportive working conditions for nurses engaged in health literacy promotion. Meanwhile, partnerships have been developed with external systems—including other healthcare institutions, community organizations, health-related enterprises, media, and universities—to extend the reach of health literacy efforts beyond individual patients to the broader population. This category includes two subcategories: collaboration among internal systems and cooperation with external systems.

#### 3.5.1. Collaboration Between Internal Systems

The nursing system actively collaborated with other intra-organizational subsystems to improve the institutional conditions necessary for effective health literacy promotion. For instance, close coordination with physicians ensured that patients with low health literacy were prioritized during interdepartmental transfers or clinical team handovers. Handover protocols included documentation of patients' health literacy levels, specific information needs, and preferred learning modalities.

Additionally, partnerships with information technology, logistics, and communications departments supported the development of digital and material infrastructure. These collaborations facilitated the co-creation of health education materials, enhanced nursing information systems, and optimized digital platforms that support patient care and communication.

**P9** (interview)*: “When encountering patients with low health literacy, after collecting their medical history, I inform the doctor that this patient requires extra attention.”*

**P7** (interview)*: “Completed health education materials or videos are shared with doctors for feedback and suggestions.”*

**P1** (interview)*: “It's best to collaborate with IT to integrate health literacy assessment tools into the nursing information system.”*

**F1** (field investigation, observation mobile application program)*: “Placing the ‘PICC Service Introduction' and ‘Appointment Booking' buttons on the same interface allows patients to book appointments immediately after viewing the service information.”*

#### 3.5.2. Cooperation With External Systems

The nursing administrative department has also established collaborations with external partners, such as community organizations, health-related enterprises, media outlets, and academic institutions, to broaden the impact of health literacy initiatives. These partnerships have expanded health literacy promotion beyond individual patients to broader populations, including diverse community groups.

**P20** (interview): *“After obtaining informed consent from patients with pressure ulcers, our department directly purchased more effective pressure ulcer dressings from partner organizations, allowing patients to use them right here in our hospital.”*

**P7** (interview)*: “The partnership between the hospital and universities is mutually beneficial. We provide clinical environments and data for research, while they have professional researchers who guide us and help us apply for health promotion-related projects.”*

**P8** (interview)*: “Recently, our endocrine head nurse went to the community to teach residents with diabetes how to inject insulin and provided free health consultations.”*

#### 3.5.3. Nursing Quality Improvement

The nursing administrative department consistently implements quality improvement initiatives aimed at enhance health literacy–related services. These projects are guided by feedback from nurses and patients regarding obstacles in accessing health information and nursing care. The effectiveness of each intervention is evaluated using suitable tools or metrics, and processes are refined based on the outcomes to ensure continuous improvement.

**P14** (interview): “*Patient feedback and suggestions are important. We distribute surveys and conduct follow-ups to see which aspects of their experiences need optimization or improvement*.”

**P7** (interview): “*We conducted a quality control circle (QCC) project to improve diabetes patients' dietary knowledge awareness rate. Measures included forming an education team, compiling easy-to-understand educational materials, and launching a WeChat account to share dietary knowledge. After one month, the awareness rate increased, and we continued using this process in our department*.”

### 3.6. Conceptual Framework of HLNS and Its Concept Definition

**Figure 2:**
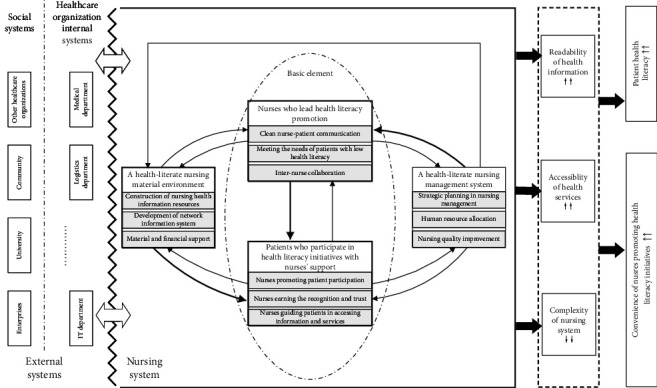
Conceptual framework of the health-literate nursing system.

Based on the analysis of relationships among the main categories, a conceptual framework for system-level nursing approaches to address health literacy needs was developed and defined as the HLNS (Figure 2).

Within this framework, four interrelated subsystems were identified. “A health-literate nursing material environment” and “a health-literate nursing management system” serve as structural supports, providing the organizational foundation for health literacy initiatives. Meanwhile, “nurses who lead health literacy promotion” and “patients who participate in health literacy initiatives with nursing support” represent the core functional subsystems, directly involved in implementing health literacy practices. These four subsystems are functionally distinct yet interdependent, continuously interacting and evolving to generate health literacy–oriented nursing support.

The “health-literate external system” acts as an external influencing subsystem that dynamically interacts with the internal components of the HLNS. It functions as a contextual driver that enhances synergy and broadens the impact of health literacy efforts.

Grounded in interview and field data, HLNS is an integrated nursing system that combines supportive environments, organizational infrastructure, and human resources to enhance the accessibility of nursing-related health information and simplify nursing services. It aims to meet diverse health literacy needs and support nurses in delivering health literacy–oriented care through four coordinated subsystems and the synergistic influence of external systems.

## 4. Discussion

To our knowledge, this study is the first to conceptualize system-level approaches for health literacy needs within the nursing system—positioning nursing as a unique subsystem with its own organizational infrastructure, workflows, and interprofessional interfaces. Drawing on empirical data, the HLNS was introduced, a system-level framework delineating how nursing practice and management can jointly address patients' health literacy needs. HLNS specifies nurses' roles in facilitating equitable access to health information and services and offers structural guidance for nursing administrators to embed health literacy into care delivery.

HLNS aligns with several established system-level concepts and frameworks aimed at reducing healthcare complexity, including the HLHO [14], Org-HLR [15], and the OHL concept embedded in Healthy People 2030 [1]. These approaches emphasize integrating health literacy into healthcare delivery through structural reform.

HLNS demonstrates conceptual compatibility with four key aspects: (1) HLNS, like HLHO, Org-HLR, and OHL, reflects a paradigm shift from viewing health literacy as an individual responsibility to a systemic function. All emphasize the need for healthcare organizations to proactively adapt to the literacy needs of diverse populations. (2) Each framework highlights the importance of leadership commitment, workforce capacity-building, and institutionalizing health literacy principles in policy and practice. (3) Effective communication is central, with HLNS highlighting “clear nurse–patient communication,” and HLHO and Org-HLR advocating plain language, comprehension checks, and tailored messages for different audiences [17, 37, 38]. (4) All advocate for reducing healthcare complexity by improving service accessibility, streamlining navigation, and providing user-friendly information resources.

Despite these conceptual similarities, HLNS introduces four distinctive contributions: (1) It defines nursing as a semiautonomous subsystem within healthcare, allowing for a targeted, profession-specific approach to health literacy that is not fully addressed in broader system-level frameworks. (2) It highlights the dual role of nurses as educators and system navigators—positioning them as frontline actors who coordinate care, advocate for patients with low health literacy, and provide feedback for system improvement [39]. (3) HLNS emphasizes the development of practical, context-specific health education tools aligned with nursing workflows, such as those for discharge planning, rehabilitation, and self-management. It also integrates digital innovations, such as networked information systems and mHealth platforms [40, 41], to support literacy efforts in increasingly tech-enabled care settings. (4) Unlike HLHO or OHL, HLNS does not address navigational aids (e.g., signage or spatial tools) [42, 43], as these typically fall outside the nursing domain. Additionally, it treats departments like logistics, IT, and media as external collaborators. These distinctions reflect HLNS's deliberate focus on the nursing subsystem, enhancing its operational specificity and applicability in nursing management.

The effectiveness of HLNS depends on institutional commitment to health literacy. In China, its implementation faces several interrelated challenges: (1) Limited patient trust in nurses. Public trust in healthcare professionals remains low, with one survey reporting an average score of 6.33 out of 10 [44]. Given that trust is fundamental to behavior change [45], this presents a significant barrier to health literacy promotion. The HLNS subcategory “nurses earning recognition and trust” highlights the need for nurses to strengthen their clinical competence and communication skills to build patient confidence [46]. (2) Inadequate institutional infrastructure. HLNS relies on broader adoption of frameworks such as HLHO and OHL, which support organizational responsibility for health literacy. However, most hospitals in China lack standardized protocols for assessing patient literacy, limiting nurses' ability to deliver tailored interventions [47–49]. Furthermore, continuity of care is often limited to nurse-led strategies such as phone calls or social media due to constrained institutional resources [50]. This fragmented infrastructure presents a key barrier to implementing system-level, literacy-sensitive nursing practices. (3) Nursing workforce shortages. With only 40 registered nurses per 10,000 people in 2023—compared to 103 in Canada, 118.8 in the United States, and 91.5 in the United Kingdom [51, 52]—China faces significant workforce constraints. Nurses often assume dual roles, including that of health education coordinators, limiting the feasibility of specialized literacy support.

This study has several limitations that inform future research directions: (1) The analysis was based solely on data from healthcare professionals, without formally including patient perspectives. Although some patient-related insights were captured through field observations—such as barriers encountered in accessing health information and nursing services—they were limited in depth and scope. Consequently, the findings primarily reflect an organizational perspective and may not fully represent patients' diverse health literacy needs. Future studies should incorporate patient voices to ensure a more balanced and comprehensive understanding [53, 54]. (2) While the interviews included nurses and managers from community health centers, field observations were conducted only in one tertiary and one secondary hospital. Therefore, the applicability of the HLNS framework in community-based healthcare settings requires further examination. (3) Health literacy research has evolved from individual interventions [55] (demand-side health literacy) to advocating organizational changes [1] to reduce the complexity of health information and services (supply-side health literacy). Although this study focused on the latter aspect, it may not fully address the varied health literacy needs of service recipients. Future research should consider both supply and demand perspectives.

## 5. Conclusions

This study conceptualizes system-level nursing approaches for addressing health literacy needs as the HLNS. This system is characterized by a nursing team centered on health literacy–oriented support. A supportive environment that enhances health literacy within nursing practice involves a team implementing improvement measures from a system-level construction perspective. This includes emphasizing nurses' leadership, promoting active patient participation, developing nursing materials, improving nursing management, and fostering external collaboration. In such an environment, patients' health literacy needs will be effectively met, and nurses' initiatives in health literacy will be conveniently implemented.

## 6. Implications for Nursing Management

The conceptual framework of HLNS developed in this study has broad potential applications. Policymakers may use this information in health system reforms. Health and social service organizations can integrate it with HLHO or OHL to improve quality and address health literacy needs. Nursing managers and clinical nurses can apply HLNS to guide health education practices, particularly in addressing the health needs of patients with low health literacy. In fact, it has been successfully implemented to develop an organizational self-assessment tool to help a nursing system evaluate its performance according to HLNS standards and plan health literacy–related improvement activities.

## Figures and Tables

**Table 1:** Participant demographic information (*n* = 23).

Participant ID	Sex	Vocation	Degree	Professional position	Work experience (year)	Institution type
P1	Female	Director of nursing department	Undergraduate	Senior	16	Hospital
P2	Female	Head nurse	Undergraduate	Senior	11	Hospital
P3	Female	Bedside nurse	Junior college	Intermediate	9	Community healthcare center
P4	Female	Bedside nurse	Master	Intermediate	13	Hospital
P5	Female	Head nurse	Master	Senior	20	Hospital
P6	Female	Director of nursing department	Master	Senior	24	Hospital
P7	Female	Head nurse	Undergraduate	Senior	15	Community healthcare center
P8	Female	Director of nursing department	Master	Senior	22	Hospital
P9	Male	Bedside nurse	Undergraduate	Intermediate	8	Hospital
P10	Female	Doctor	Master	Intermediate	14	Hospital
P11	Female	Director of nursing department	Master	Senior	21	Hospital
P12	Female	Bedside nurse	Undergraduate	Intermediate	10	Hospital
P13	Male	Doctor	Master	Senior	19	Hospital
P14	Male	Administrative manager	Doctorate	Senior	11	Hospital
P15	Male	Administrative manager	Master	Senior	23	Community healthcare center
P16	Female	Administrative manager	Master	Senior	20	Community healthcare centers
P17	Female	Health administrative government department officer	Master	Level IV division rank official	10	Health administrative government
P18	Female	Health administrative government department officer	Undergraduate	Level II principal staff member	7	Health administrative government
P19	Female	Bedside nurse	Undergraduate	Intermediate	7	Community healthcare center
P20	Female	Bedside nurse	Undergraduate	Junior	6	Hospital
P21	Female	Bedside nurse	Undergraduate	Intermediate	8	Hospital
P22	Female	Bedside nurse	Undergraduate	Junior	6	Hospital
P23	Female	Bedside nurse	Undergraduate	Junior	5	Hospital

**Table 2:** Demographic information of the field sites (*n* = 2).

Item	Hospital ID	
	F1	F2
Location	Taizhou, Zhejiang province	Ningbo, Zhejiang province
Hospital level	Secondary hospital	Tertiary hospital
Foundation year	1952	1938
Building area (m^2^)	30,000	100,000
Number of approved beds (beds)	590	1000
Number of employees (person)	750	1123
Number of medical and technical personnel (person)	525	918
Whether to carry out internet hospital construction	Yes	Yes
Whether to build a health promotion hospital	Yes	Yes

**Table 3:** Core category, main categories, and subcategories of health-literate nursing system.

Core category	Main categories	Subcategories
Health literacy–oriented nursing support	1. Nurses who lead health literacy promotion	A1. Clear nurse–patient communication
		A2. Meeting the needs of patients with low health literacy
		A3. Inter-nurse collaboration
	2. Patients who participate in health literacy initiatives with nursing support	A4. Nurses promoting patient participation
		A5. Nurses earning the recognition and trust
		A6. Nurses guiding patients in accessing information and services
	3. A health-literate nursing material environment	A7. Construction of nursing health information resources
		A8. Development of network information system
		A9. Material and financial support
	4. A health-literate nursing management system	A10. Strategic planning in nursing management
		A11. Human resource allocation
		A12. Nursing quality improvement
	5. A health-literate external system	A13. Collaboration between internal systems
		A14. Cooperation with external systems

## Data Availability

Data supporting the findings of this study are available from the corresponding author upon reasonable request.

## Supporting Information

Supporting Information 1 Supporting 1: Interview frequency, reasons for multiple interviews, and result of member checking.

Supporting Information 2 Supporting 2: Result of the theoretical saturation test.

Supporting Information 3 Supporting 3: Health-literate nursing system subcategories and main category.
