# Supplementary material for: Conceptual Framework of Health-Literate Nursing System: A Proceduralized Grounded Theory Approach
Source: J Nurs Manag. 2025 Jul 18;2025:1496009. doi: 10.1155/jonm/1496009 (PMC12297149; doi:10.1155/jonm/1496009)
Supplement: Supporting Information 1 — Supporting 1: Interview frequency, reasons for multiple interviews, and result of member checking. [file 1496009.f1.docx]

## Supplementary 1. Interview Frequency, Reasons for Multiple Interviews, and Result of Member Checking

| Participant  ID | Vocation | Interview Frequency | Member checking of first interview transcript | Second rounds interview | Third rounds interview |
| --- | --- | --- | --- | --- | --- |
| P1 | director of nursing department | 3 | minor clarify | clarify unclear statements & gather additional information | gather additional information |
| P2 | head nurse | 1 | minor clarify |  |  |
| P3 | bedside nurse | 2 | confirm | gather additional information |  |
| P4 | bedside nurse | 1 | No response |  |  |
| P5 | head nurse | 1 | minor clarify |  |  |
| P6 | director of nursing department | 2 | confirm | clarify unclear statements |  |
| P7 | head nurse | 2 | minor clarify | clarify unclear statements |  |
| P8 | director of nursing department | 1 | confirm |  |  |
| P9 | bedside nurse | 1 | confirm |  |  |
| P10 | doctor | 1 | confirm |  |  |
| P11 | director of nursing department | 1 | minor clarify |  |  |
| P12 | bedside nurse | 1 | minor clarify |  |  |
| P13 | doctor | 2 | Offer additional insights | gather additional information |  |
| P14 | Administrative manager | 1 | confirm |  |  |
| P15 | Administrative manager | 1 | No response |  |  |
| P16 | Administrative manager | 3 | minor clarify | clarify unclear statements & gather additional information | gather additional information |
| P17 | health administrative government department officer | 1 | minor clarify |  |  |
| P18 | health administrative government department officer | 1 | confirm |  |  |
| P19 | bedside nurse | 2 | Offer additional insights | gather additional information |  |
| P20 | bedside nurse | 1 | confirm |  |  |
| P21 | bedside nurse | 2 | confirm | clarify unclear statements |  |
| P22 | bedside nurse | 2 | Offer additional insights | gather additional information |  |
| P23 | bedside nurse | 1 | No response |  |  |
