# Supplementary material for: Conceptual Framework of Health-Literate Nursing System: A Proceduralized Grounded Theory Approach
Source: J Nurs Manag. 2025 Jul 18;2025:1496009. doi: 10.1155/jonm/1496009 (PMC12297149; doi:10.1155/jonm/1496009)
Supplement: Supporting Information 2 — Supporting 2: Result of the theoretical saturation test. [file 1496009.f2.docx]

## Supplementary 2. Result of the Theoretical Saturation Test

Between July to September 2024, ten healthcare professionals who were not involved in the earlier phase of this study were selected for interviews to test the theoretical saturation of the constructed concept and conceptual framework. During the saturation testing, open coding yielded a total of 71 codes, from which 5 categories were extracted: “easy to understand nurse-patient communication, “implementation of universal preventive measures for health literacy”, “patient participation in the evaluation of health information”, “software support for Internet + nursing” and “nursing cooperation with hospitals within the medical alliance group”.

All 5 categories overlap with the categories established in the earlier phase of this study. For example, the original statement, “The head nurse of our department regularly interviews patients and asks for their feedback on the department’s education manual”, was coded as “patient participation in the evaluation of health information”, which corresponds to the previously identified category “patient participation in the design and evaluation of health information”. Additionally, no new core categories or novel inter-category relationships emerged during the analysis, suggesting that the categories within the HLNS conceptual framework were sufficiently comprehensive. Therefore, it can be concluded that the HLNS conceptual framework has achieved theoretical saturation.
