# Supplementary material for: Conceptual Framework of Health-Literate Nursing System: A Proceduralized Grounded Theory Approach
Source: J Nurs Manag. 2025 Jul 18;2025:1496009. doi: 10.1155/jonm/1496009 (PMC12297149; doi:10.1155/jonm/1496009)
Supplement: Supporting Information 3 — Supporting 3: Health-literate nursing system subcategories and main category. [file 1496009.f3.docx]

## Supplementary 3. Health-literate Nursing System subcategories and main category

| Subcategories | Categories |
| --- | --- |
| A1 Clear nurse-patient communication | a1 Verbal communication between nurse and patient is understandable |
|  | a2 Nurses and patients communicate using multiple communication modalities |
|  | a3 Provide support for high-risk communication situations |
|  | a4 Implementing health literacy universal precautions |
| A2 Meeting the needs of patients with low health literacy | a5 Identify patients with limited health literacy |
|  | a6 Providing targeted assistance to patients with limited health literacy |
| A3 Inter-nurse collaboration | a7 Nurses collaborate effectively with each other |
|  | a8 Inheriting and practicing nursing culture |
| A4 Nurses promoting patient participation | a9 Patient involvement in medical decision making |
|  | a10 Patient involvement in the design and evaluation of health information |
|  | a11 Patient participation in patient outreach |
| A5 Nurses earning the recognition and trust | a12 Patients acknowledge and trust the professional competence of nurses |
| A6 Nurses guiding patients in accessing information and services | a13 guiding patients in accessing credible sources of healthcare information and services |
| A7 Construction of nursing health information resources | a14 Health information materials are easy to understand and available in diverse formats |
|  | a15 Health information materials are easy to obtain |
| A8 Construction of network information system | a16 “Internet + Nursing Services” are diverse and well-developed |
|  | a17 Network information platforms are available in multiple formats |
| A9 Material and financial support | a18 Material and financial resources allocation |
| A10 Top-level design of nursing management | a19 Nursing management defines clear job responsibilities |
|  | a20 Nursing culture formation |
|  | a21 Nursing regulations and operational systems are comprehensive |
| A11 Human resource allocation | a22 Nursing human resources deployment |
| A12 Nursing quality improvement | a23 Development and implementation of quality improvement plans |
|  | a24 The effectiveness of quality improvement initiatives is regularly evaluated |
| A13 Collaboration between internal system | a25 Nurses collaborate in caring for patients with limited health literacy |
|  | a26 Technical (software and hardware) infrastructure supports health literacy efforts |
| A14 Cooperation with external system | a27 Collaboration with other medical institutions |
|  | a28 Collaboration with non-medical institutions and organizations |
